# Silicone Oil Droplets in the Vitreous After an Anti-vascular Endothelial Growth Factor (VEGF) Injection: A Complication of Syringe Lubrication

**DOI:** 10.7759/cureus.78715

**Published:** 2025-02-07

**Authors:** Sripathi Kamath, Akshata Charlotte, Nandini Nandan

**Affiliations:** 1 Ophthalmology, Father Muller Medical College, Mangalore, IND; 2 Ophthalmology, Dr. Suresh Babu Eye Foundation, Kasaragod, IND

**Keywords:** anti-vegf vitreous injection, case report, intra-vitreal, post intra-vitreal injection, silicone oil, vitreous floaters

## Abstract

Intravitreal anti-vascular endothelial growth factor (VEGF) injections are a cornerstone treatment for various retinal conditions, including diabetic macular edema and age-related macular degeneration. While generally safe, these injections can introduce unintended substances, such as silicone oil droplets, into the vitreous cavity due to the silicone-based lubricant used in syringe manufacturing. Although frequently asymptomatic, silicone oil droplets can occasionally cause significant visual disturbances and discomfort. We report a rare case of symptoms caused by silicone oil droplets following a single intravitreal bevacizumab injection.

A 54-year-old diabetic male patient presented with pain and iridescent floaters in his right eye, which began immediately after receiving an intravitreal bevacizumab injection and persisted for four weeks. The patient, with a 20-year history of diabetes and prior scatter laser photocoagulation, exhibited a visual acuity of 20/80 in the right eye or oculus dexter (OD) and 20/40 in the left eye or oculus sinister (OS), with an elevated intraocular pressure (IOP) of 24 mmHg in the right eye. Dilated fundoscopy revealed multiple silicone oil droplets floating in the vitreous cavity, alongside severe non-proliferative diabetic retinopathy and macular edema. The patient was started on anti-glaucoma medications, brimonidine, and timolol, which effectively reduced his IOP to 18 mmHg within a week. Despite IOP control, the patient remained distressed by persistent floaters, which gradually became less symptomatic with time.

This case is unique because the symptoms due to the silicone oil droplets occurred after a single injection, unlike most reports of asymptomatic presentations or those arising after multiple injections. The disproportionate amount of silicone oil observed raises concerns about syringe design and transport conditions. While silicone oil is inert, its presence can cause the patient anxiety and visual disturbances.

## Introduction

Intravitreal anti-vascular endothelial growth factor (VEGF) injections are widely used to treat various retinal conditions, including diabetic macular edema, age-related macular degeneration, and retinal vein occlusions. However, a notable complication observed with this procedure is the presence of silicone oil droplets in the vitreous cavity. Studies have shown that silicone oil droplets were present in 71% of patients receiving intravitreal anti-VEGF injections, although only 40% of these individuals reported experiencing visual symptoms [1]. This discrepancy suggests that many cases may remain asymptomatic, leading to underreporting or undiagnosed occurrences.

The silicone oil droplets originate from the silicone-based lubricant that coats the syringe barrel and plunger, facilitating smoother and more efficient drug delivery. The polymer most commonly used for this purpose is polydimethylsiloxane, a form of silicone known for its low surface energy and hydrophobic properties. While its presence improves the functionality of syringes, it also contributes to the inadvertent introduction of silicone oil into the eye during intravitreal injections.

The occurrence of silicone oil droplets appears to be influenced by several factors, with a notable trigger being the common practice of flicking the syringe before the injection [2]. This action, intended to eliminate air bubbles and prime the syringe, has been implicated in the release of silicone oil into the vitreous cavity. Additionally, a correlation has been observed between a higher number of silicone oil droplets and an increase in mean IOP, raising concerns about potential long-term complications [1].

Despite global reports documenting the presence of silicone oil droplets post-intravitreal injections, the incidence of this phenomenon in South Asian populations remains largely unstudied. We present a case of multiple silicone oil droplets in a 54-year-old diabetic patient following an intravitreal injection of bevacizumab. This case highlights the need for further investigation into regional variations, potential risk factors, and clinical implications associated with this phenomenon.

## Case presentation

A 54-year-old diabetic male patient presented with complaints of pain and iridescent floaters in the right eye. His symptoms worsened when he looked at bright lights. He presented to us four weeks after receiving an intravitreal bevacizumab injection to the right eye. However, his onset of symptoms was one week after receiving this intravitreal injection. The symptoms did not increase or decrease in intensity during this period. He is a known Type 2 diabetic patient who has been on insulin therapy for the last 20 years, which was fairly controlled with a recent HbA1c of 6.9%. He has also been hypertensive for the past 10 years. He had undergone scatter laser photocoagulation therapy in both eyes 10 months ago to address severe non-proliferative diabetic retinopathy (NPDR). The patient had no history of ocular infections, such as uveitis. There was no history of high myopia. He also denied experiencing flashes of light, and there was no history of recent eye trauma that could contribute to the development of floaters. The patient's pre-procedure visual acuity was 20/60 in the right eye (oculus dextrus or OD) and 20/40 in the left eye (oculus sinister or OS), with intraocular pressures (IOP) of 14 mmHg and 12 mmHg, respectively. However, his visual acuity post-procedure was 20/80 OD and 20/40 OS, accompanied by an elevated IOP of 24 mmHg (reference range: 10-21 mmHg) in the right eye, while that of the left eye remained stable at 12 mmHg. A detailed dilated fundoscopy of the right eye with a +90 D lens (Volk Optical, Inc., Mentor, USA) revealed multiple oil droplets of various sizes floating in the vitreous medium, moving along with eye movement.

**Figure 1:**
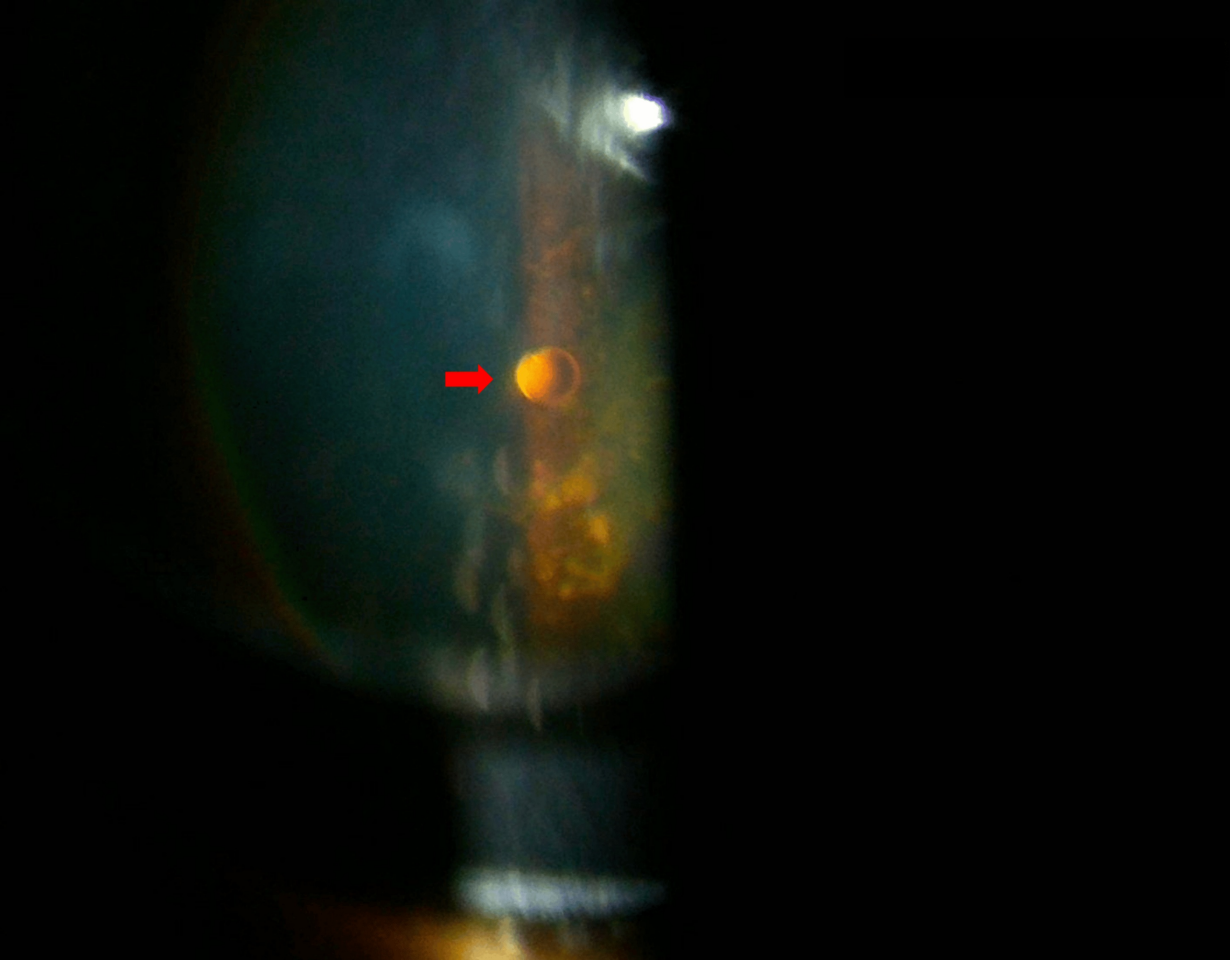
Slit lamp biomicroscopy photograph with +90D lens showing a silicon oil bubble floating in the mid vitreous, highlighted by the red arrow

Figure 1 shows the slit lamp biomicroscopy photograph with a silicon oil bubble floating in the mid-vitreous.

**Figure 2:**
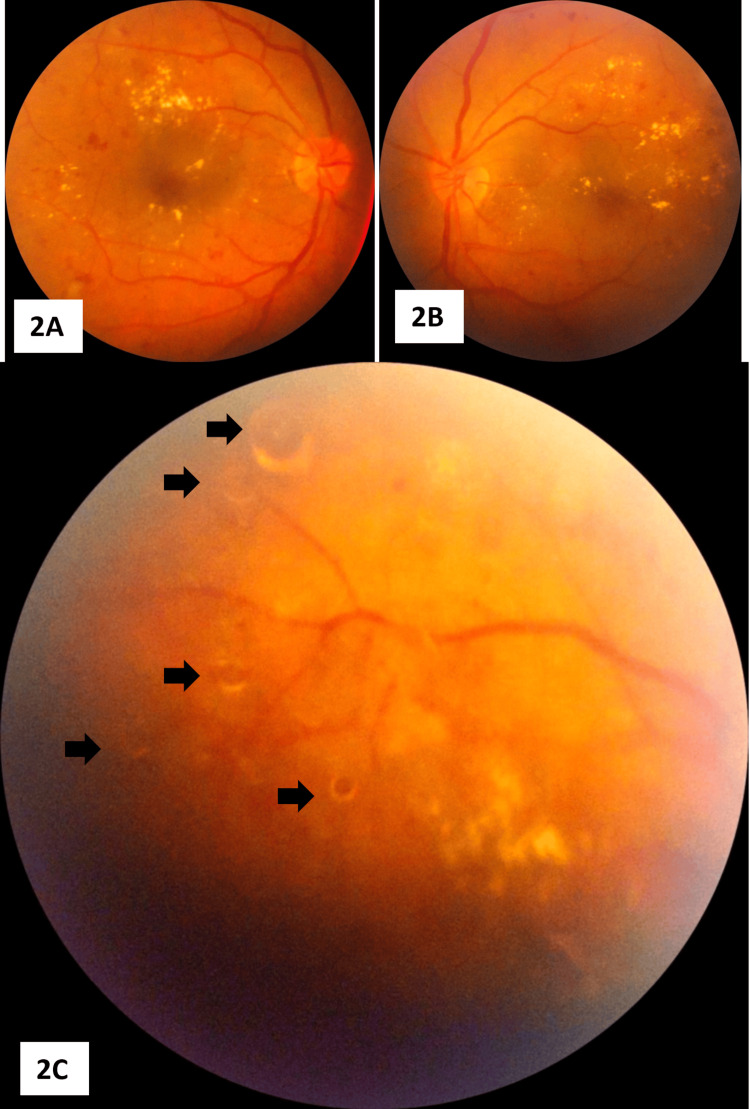
Fundus images of both eyes 2A: Fundus photograph of the right eye shows severe non-proliferative diabetic retinopathy (NPDR) changes, microaneurysms, hard exudates, and diabetic macular edema with post-scatter laser photocoagulation scars. 2B: Shows the left eye fundus, also with severe NPDR changes(OD>OS). 2C: Focuses on a mid-peripheral fundus region of the right eye, showing background severe diabetic retinopathy changes with multiple silicone oil droplets of varying sizes floating in the posterior hyaloid phase, highlighted by the black arrows. OD: Right eye, OS: Left eye

The fundus images (Figures 2A-2C) showed a background retina with post-scatter laser photocoagulation scars, severe NPDR changes, microaneurysms, hard exudates around the macula, and macular edema.

The patient was promptly started on two anti-glaucoma medications, brimonidine and timolol, both administered twice daily to manage the elevated IOP in the right eye. He was advised to avoid activities that could exacerbate symptoms, such as exposure to bright lights, and asked to return for follow-up after one week.

At the one-week follow-up, the patient reported a significant reduction in ocular pain and discomfort, indicating an effective IOP-lowering response to the prescribed medications. The IOP in the right eye had decreased to 18 mmHg, demonstrating good control. Despite this improvement, the patient remained distressed about the persistence of iridescent floaters.

During the follow-up examination, dilated fundoscopy confirmed the presence of silicone oil droplets in the vitreous cavity, with no evidence of new droplets or additional complications, such as retinal detachment or hemorrhage. The patient was reassured that the floaters were non-threatening and would likely become less noticeable over time as the brain adapts to ignore them through a process known as neuroadaptation. He was also educated about the benign nature of silicone oil droplets and reassured that invasive interventions, such as vitrectomy, were not warranted given the stable clinical picture and absence of significant visual deterioration. 

The patient was followed up over four months, with initial fortnightly follow-ups for the first month, followed by monthly follow-ups thereafter. By the third month, the patient's discomfort related to floaters had significantly diminished, and his IOP remained stable under medical management. While the floaters persisted, the patient reported better psychological coping and improved quality of life. 

## Discussion

Our case highlights a rare complication of symptoms due to silicone oil droplets in the vitreous cavity following an intravitreal bevacizumab injection. Despite being non-threatening, these droplets caused significant discomfort and anxiety to the patient. His long-standing diabetes, history of pan-retinal photocoagulation, and elevated IOP added complexity to the presentation. Early identification, effective IOP management, and patient counseling were key to alleviating symptoms and improving psychological well-being.

Intravitreal injections are one of the most common ophthalmic procedures. Intravitreal anti-VEGF injections are routinely being used to treat various ocular diseases such as diabetic macular edema, choroidal neovascularization associated with wet age-related macular degeneration, pathological myopia, retinopathy of prematurity and macular edema due to retinal vascular occlusions [3]. There has been a rise in the number of intravitreal anti-VEGF injections due to the increasing diabetic population and the geriatric age group [4]. Side effects due to intravitreal injections include a transient rise in the IOP [5], inflammatory adverse effects such as retinal vasculitis, sterile intraocular inflammation, and post-intravitreal injection endophthalmitis [6]. Injection site complications, such as subconjunctival hemorrhage and conjunctival chemosis, are well established. Most patients receiving intravitreal anti-VEGF injections may require multiple doses spaced weeks or months apart. Along with delivering anti-VEGF drugs through intravitreal injections, other unintended substances like silicone oil can enter the eye. Silicone oil is commonly used to coat the inside of syringe barrels to ensure smoother drug delivery. However, this complication is rarely reported [7].

Silicone oil droplets are introduced into the vitreous cavity during intravitreal injections as these syringes are sprayed with a silicon polymer (polydimethylsiloxane or PDMS) to reduce friction between the plunger and barrel. These oil droplets can vary in size and quantity, with a moderate number of droplets being more likely to cause symptoms, as documented in previous studies [1,8]. However, most reported cases involve asymptomatic presentations or symptoms appearing after multiple injections. The incidence of silicone oil droplets increases with the number of injections [2], but in this case, symptomatic floaters were noted immediately after the first injection. This may have occurred due to preexisting structural abnormalities in the vitreous, such as early posterior vitreous detachment, which could have facilitated the dispersion of silicone oil droplets. Additionally, the altered light scattering from the droplets, combined with the patient’s increased sensitivity to visual disturbances, may have amplified the symptomatic perception of the floaters.

The American Academy of Ophthalmology’s 2018 survey revealed that 60.4% of American and 27% of international ophthalmologists had observed silicone oil droplets in their patients, though only a small percentage reported symptomatic cases that required interventions such as vitrectomy (1.4% in the US, 5.2% in international studies) [3]. This highlights the unusual nature of the current case where symptoms arose after just one injection.

The distribution of droplets in the vitreous cavity depends on the consistency of the vitreous gel, the injection technique, and buoyancy dynamics. In younger individuals or those with an intact vitreous, the gel-like structure provides a supportive matrix that can trap droplets in the mid-vitreous, whereas, in older individuals with a liquefied or partially detached vitreous, injected substances disperse more freely and may rise to the top due to buoyancy [9]. The interaction between the gel and liquid phases of the vitreous plays a crucial role in determining droplet movement [10]. The injection technique also influences droplet distribution, as a slow, deep injection into the gel matrix may lead to entrapment within vitreous strands. In contrast, a rapid, high-force injection can create turbulence and disperse droplets more widely [9,10]. Buoyancy is another key factor, as the relative density of the injected droplets compared to the surrounding vitreous determines their movement. However, in an incompletely liquefied vitreous, droplets may remain suspended rather than floating upward [10]. In our case, the droplets were observed in the mid-vitreous, likely due to a partially liquefied vitreous with sufficient gel support to prevent immediate ascent while still allowing some degree of suspension.

Another unique aspect of this case was the disproportionate amount of silicone oil observed in the vitreous cavity compared to the volume of the drug administered. While silicone oil is generally inert, it acts as a foreign body in the eye and can exacerbate inflammatory responses, leading to persistent floaters. Silicone oil is known to remain in the eye for 12 to 18 months, with the potential to become trapped in the trabecular meshwork, thereby increasing IOP [1,11]. Numerous studies have reported a mean increase in IOP in the eyes with silicone oil droplets, although this increase is often not statistically significant [1]. But our patient had increased IOP, most probably due to the volume of the bubbles present.

The excessive amount of silicone oil may be attributed to potential defects in the syringe design, leading to the unintentional release of silicone oil coating during the injection process. It is documented that though it is almost impossible to inject and reproduce the same amount of anti-VEGF, syringes with low dead space and the shortest possible stroke length can be used to reduce silicon abrasion on the inner walls of syringes [3].

Interestingly, though common practices like flicking the syringe before injection can dislodge the silicone oil, the syringe in this case was not flicked. Yet, a substantial amount of silicone oil entered the eye. This can be attributed to manufacturing defects in syringes. These defects include uneven siliconization during syringe production, which can lead to the detachment of silicone oil droplets from the inner walls or plunger of the syringe during injection. Such droplets are introduced into the administered solution, posing a risk of contamination and adverse ocular outcomes [12].

Additionally, in our patient, suboptimal storage and transport conditions such as exposure to fluctuating temperatures and mechanical agitation could have degraded the silicone layer, making it more susceptible to migration into the administered solution. Repeated freeze-thaw cycles during storage or transport can dislodge silicone oil droplets from the syringe walls into the medication. Studies have shown that freezing syringes preloaded with protein formulations significantly increases the silicone oil droplet count, which can compromise the medication's quality and safety​. Mechanical agitation, such as end-over-end shaking during transport, can lead to the formation of protein-silicone oil complexes and increased particle counts. These interactions occur at the silicone oil-water interface, exacerbating contamination risks. Sudden mechanical shocks, such as dropping syringes or shipping boxes, can cause silicone oil droplets to detach from the syringe walls and mix into the solution. This has been observed to significantly elevate silicone oil contamination levels and may damage protein therapeutics through cavitation effects. Exposure to temperatures above recommended storage limits promotes protein unfolding, aggregation, and interaction with silicone oil droplets. The resulting degradation reduces the therapeutic efficacy and safety of the medication [12].

Furthermore, the specific syringe design used may have contributed to this outcome. Syringes employing a staked-on needle design, as opposed to luer lock designs, often provide less robust retention of the silicone lubricant, thereby increasing the risk of contamination. A study comparing different syringe types found that syringes with staked-on needles released more silicone oil droplets upon agitation, highlighting the influence of syringe design on contamination [11]. Additionally, pre-filled syringes have shown the lowest number of particles compared to repackaged drugs in staked-in needle syringes, suggesting that syringe design plays a critical role [3]. Raising awareness about the routine coating of syringes with silicone and the potential for unintended release of silicone oil into the eye is essential to mitigate this complication.

In rare cases, if the floaters cause significant visual loss, vitrectomy becomes the treatment of choice. However, this invasive procedure carries risks such as postoperative endophthalmitis, retinal tears, retinal detachment, and vitreous hemorrhage [13].

This case is distinct from previously reported instances due to several notable factors. Firstly, the patient developed symptoms due to the floaters of silicone oil droplets immediately after the first intravitreal bevacizumab injection, whereas most documented cases involve asymptomatic presentations or symptoms arising after multiple injections. Secondly, the quantity of silicone oil observed in the vitreous cavity was disproportionately larger than the volume of the drug administered. Lastly, unlike common practices that involve flicking the syringe which is known to dislodge silicone oil, the syringe was not flicked before injection, yet a substantial amount of silicone oil was introduced. These unique aspects heighten the need for further investigation into syringe design, handling techniques, and manufacturing quality to mitigate such complications.

## Conclusions

In conclusion, ophthalmologists can take several practical steps to minimize the risk of silicone oil contamination during intravitreal injections. First, the use of prefilled syringes may reduce the need for manual manipulation and plunger friction, thereby lowering the likelihood of dislodging silicone oil droplets. Additionally, selecting syringes that employ minimal silicone lubrication or are specifically designed to minimize oil release can further reduce contamination risks. Finally, avoiding unnecessary priming or flicking of the syringe can help preserve the integrity of the lubricant layer, ensuring a safer injection process. These strategies collectively contribute to a safer, more controlled clinical environment for patients undergoing intravitreal procedures.
